# A novel fermentation process for low salt shrimp paste based on microbial diversity and physicochemical properties analysis

**DOI:** 10.1016/j.fochx.2025.102465

**Published:** 2025-04-16

**Authors:** Meiqi Gu, Ting Li, Pinghui Pan, Aneth Massawe, Chuandong Fang, Chuanhai Tu, Zhiyu Liu, Bin Zhang

**Affiliations:** a College of Food Science and Pharmacy, Zhejiang Ocean University, Zhoushan 316022, PR China; b College of Biosystems Engineering and Food Science, Zhejiang University, Hangzhou 310058, PR China; c Zhejiang Xingye Group Co. Ltd., Zhoushan 316000, PR China; d Key Laboratory of Cultivation and High-value Utilization of Marine Organisms in Fujian Province, Fisheries Research Institute of Fujian, Xiamen 361013, PR China

**Keywords:** Low-salt shrimp paste, Fermentation process, Carbon source, Microbial diversity, High-throughput sequencing

## Abstract

This study investigated the changes in physicochemical properties and microbial diversity during the fermentation of low-salt shrimp paste with the addition of different carbon sources (glucose and glutinous rice flour). Notably, during fermentation, the low-salt shrimp paste with glucose and glutinous rice flour had lower TVB-N values and higher amino acid nitrogen (AAN) values, which may be responsible for increasing the flavor of the low-salt shrimp paste compared to the control group. Addition of glucose and glutinous rice flour to low-salt shrimp paste is beneficial in inhibiting histamine production. High-throughput sequencing showed that at the family level, the relative abundance of Bacillaceae at the end of fermentation was 63.89 % in the control group, 57.31 % in the group with the addition of glucose, and 44.18 % in the group with the addition of glutinous rice flour. The addition of glucose and glutinous rice flour reduced the relative abundance of Bacillaceae, which were partially pathogenic. Changes in the physicochemical properties were associated with various microorganisms, which collectively impacted the quality of low-salt shrimp paste. This study provides a theoretical reference for improving the quality of shrimp paste, reducing the cost of shrimp paste, and further advancing the fermentation process of novel low-salt shrimp paste.

## Highlights

• Additional carbon sources significantly affected the microbial dynamics.
• The addition of different carbon sources can regulate microbial flora.
• The addition of glucose and glutinous rice flour reduced histamine content.
• Glucose and glutinous rice flour enriched the flavor of low-salt shrimp paste.

## 1 Introduction

Known for its distinct flavors and texture, shrimp paste is a classic fermented delicacy popular in Eastern and Southeast Asian coastal regions (Yu et al., 2022). The biological advantages of shrimp paste, abundant in polyunsaturated fatty acids (PUFA), peptides, and amino acids, encompass antibacterial properties, lowering blood pressure and cholesterol, combating thrombosis, and antioxidant attributes (Kleekayai et al., 2015). However, the traditional shrimp paste is mostly naturally fermented, and its salt content is generally 25 % ∼ 30 %, although it can be better prevented from rotting and deterioration, and is convenient for storage, the high salt content leads to the poorer flavor of shrimp paste, the long-term consumption of shrimp paste is prone to cause cardiovascular diseases of the organism, and even lead to cancer (He & MacGregor, 2009). Hence, the traditional shrimp paste is not in line with the current lifestyle of people's healthy diet.

Low-salt shrimp paste has a salt content of only 10 % ∼ 12 % (*w*/w), which is significantly lower than that of typical shrimp paste (Li et al., 2023). Consequently, consuming low-salt shrimp paste can significantly decrease salt consumption, aligning better with the public's demand for a nutritious diet. Low-salt shrimp paste due to its lower salt addition, microorganisms grow rapidly, and fermentation time is shortened, which is good for production. Nonetheless, reduced salt levels could potentially cause spoilage, accelerating the proliferation of detrimental microbes. Additionally, the taste and quality of low-salt shrimp paste are prone to instability owing to climatic variations. Therefore, controlling the growth of harmful microorganisms during the fermentation of shrimp paste and improving flavor becomes especially important. Extant studies focused on the fermentation temperatures and the composition of the bacterial community of low-salt shrimp paste (Li et al., 2022; Yang et al., 2023). Granados-Arvizu et al. (2019) investigated the effect of glucose, xylose, and arabinose as carbon sources on the growth and fermentation of *Scheffersomyces stipitis* at three levels of agitation (0, 125, and 250 rpm). Nguyen et al. (2024) discovered that incorporating carbon sources enhanced water quality, maintained a high carbon-to‑nitrogen ratio, and augmented bacterial communities in sediments, thereby boosting marron's growth and immune efficacy. Rice flour and sugar are effective in altering the fermentative flora and improving the quality of some fermented meat food, such as sour meat and Isan sausage (Honghtong et al., 2020; Lu et al., 2022; Zhang et al., 2020). Therefore, carbon sources including glutinous rice flour and glucose were added to low-salt shrimp paste for achieving the purpose of controlling microbial flora and increasing product quality. In recent years, the technique of high-throughput sequencing (HTS) has been extensively used in examining the variety of microbes in fermented edibles, including fermented vinasse hairtail and mandarin fish. (Ding et al., 2024; Zhang et al., 2023).

In this study, glucose and glutinous rice flour were added to low-salt shrimp paste as carbon sources to study the changes in physicochemical properties and bacterial communities during fermentation. This study helps to elucidate how different carbon sources regulate the bacterial flora structure in low-salt shrimp paste, thereby enhancing the fermentation quality of shrimp paste, and providing a theoretical reference for developing new shrimp paste products.

## 2 Materials and methods

### 2.1 Samples collection

Fresh red shrimp (*Solenocera crassicornis*) weighing an average of about 15 ± 5 g were purchased from Zhoushan International Aquatic City, Zhoushan, China. To maintain the samples' freshness, they were promptly transferred to the laboratory within one hour using a foam box equipped with ice packs. Salt and glutinous rice flour were purchased from local supermarkets in Zhoushan City, Zhejiang Province, China, and food-grade glucose was purchased from Want Want Food Additives Co.

### 2.2 Fermentation of low-salt shrimp paste

Firstly, the surface of fresh shrimp was rinsed with sterile water, drained, pulped for 3 min, and put into the fermentation tank, 250 g per tank, then divided into three groups: group Y (add salt of 12 % of shrimp body mass), group P (add salt of 12 % of shrimp body mass with 6 % of glucose, and group F (add salt of 12 % of shrimp body mass with 6 % of glutinous rice flour), and group Y was the control group, stirred well and sealed with sterile gauze. The samples were placed in an incubator at 25 °C for 12 days, which were taken every three days for measurement. Microbial community analysis using high-throughput technology was performed by selecting different stages of fermentation (early, middle, and late stages) for analysis, with samples collected every six days for measurement.

### 2.3 Analysis of texture profiles analysis (TPA) and alterations in color

Evaluation of the low-salt shrimp paste samples was conducted with a TA.XT PlusC texture analyzer (Stable Micro Systems Ltd., Godalming, UK). Configuration settings for the texture analyzer included a 0.05 N trigger force, 30 % compression depth, and a compression duration of 2 s. To verify the precision and dependability of the gathered data, every measurement set was replicated six times.

Measurements of L* (lightness), a* (green-red color), and b* (blue-yellow color) were conducted using a CS-210 colorimeter produced by Hangzhou CHNSpec Technology Co., Ltd. Located in Hangzhou, China. Using Faithong's method, measurements were taken three times on each side of each sample at three different locations and repeated three times for each condition.

### 2.4 Examination of the physicochemical properties

To determine the pH value of the low-salt shrimp paste, a mixture of 5 g of the paste and 10 mL of distilled water was prepared. The blend underwent intense agitation for three min, succeeded by a five-minute interval of rest at ambient temperature. The pH value was measured utilizing a digital pH meter (PHS-3C, Shanghai YiDian Scientific Instrument Co. Ltd., Shanghai, China). The measurement of total volatile basic nitrogen (TVB-N) was conducted using Kay-type nitrogen equipment (K9840, Shandong Haineng Scientific Instrument Co., Ltd., Shandong, China) (Sang et al., 2020). AAN was determined by using the amino acid nitrogen detection kit produced by Nanjing Maogai Microbiology Technology Co. The measurement of malonaldehyde (MDA) concentrations in samples of low-salt shrimp paste was conducted following the method described by Xu et al. (2022). Moisture content and total acid were measured following the Chinese Standard GB 5009.3–2016a (2016), and Chinese Standard GB 12456–2021 (2021), respectively.

### 2.5 Analysis of amino acids and determination of histamine and water activity

The amino acid content in low-salt shrimp paste was analyzed according to Chinese Standard GB 5009.124–2016b (2016). A freeze-dried low-salt shrimp paste sample (1.5 g) was added to 10 mL of 6 mol/L HCl solution, filled with nitrogen, and hydrolyzed at 110 °C for 22 h. The solutions underwent centrifugation, precipitation, and drying by an evaporator. To dissolve the acquired residue, 2 mL of sodium citrate buffer solution was introduced, followed by an analysis of the amino acid (AA) composition using an automated amino acid analyzer (LA 8080, HITACHI, Tokyo, Japan) post-filtration through a 0.22 μm membrane.

Histamine in shrimp paste was detected by enzyme linked immunosorbent assay (ELISA) (Diniz et al., 2021), using a shrimp histamine detection kit (Jiangsu Enzyme Immunity Industry Co.). Weigh 1 g sample, add 9 mL PBS with pH 7.2–7.4, and fully homogenize. Centrifuge for about 20 min (2000 rpm) and carefully collect the supernatant. The standards in the kit were diluted to 24 μg/L, 12 μg/L, 6 μg/L, 3 μg/L, and 1.5 μg/L. The standard was accurately spiked with 50 μL in the enzyme plate, and 40 μL of sample dilution was added first to the sample wells to be tested, followed by 10 μL of the sample to be tested. The plates were sealed and incubated at 37 °C for 30 min. Discard the liquid, spin-dry, fill each well with washing solution, let it stand for 30 s and then discard it, and so on 5 times. Add 50 μL of enzyme labeling reagent to each well, except the blank wells. Temperature incubate again for 30 min. Wash again 5 times. Add 50 μL of colorant to each well and develop the color at 37 °C for 10 min away from light. The reaction was terminated by adding 50 μL of termination solution per well. The absorbance (OD value) of each well was measured sequentially at 450 nm by zeroing with blank wells. The concentration of histamine in shrimp paste was calculated by standard curve. Water activity was measured following the Chinese Standard GB 5009.238–2016c (2016).

### 2.6 Extraction of DNA, amplification via PCR, and sequencing

During the fermentation phase, samples of low-salt shrimp paste were gathered on days 0, 6, and 12, serving as representative samples. The sample was combined with sterile water in a ratio of 1:9 within a sterile conical flask. Subsequently, the mixture was placed in a shaker and incubated at a speed of 300 rpm for 1 h. Following the incubation period, the solution was passed through three layers of sterile gauze using a filtration process. Following this, the purified mixture underwent centrifugation for 15 min at a force of 12,000 ×g and a temperature of 4 °C. The precipitate, which encompassed the microbial communities, was subsequently utilized for further analysis.

Genomic DNA extraction was performed utilizing the FastDNA Spin kit for soils, a technique supplied by MP Biomedicals in Norcross, GA, USA. The V3-V4 hypervariable segments of the 16 SrRNA gene were magnified through PCR, employing universal primers 338F (5′-ACTCCTACGGGGAGGCAGCAG-3′) and 806R (5′-GGACTACHVGGGTWTCTAAT-3′) to amplify the V3-V4 region of the 16 SrRNA gene, aiding in bacterial community studies. Amplification of the ITS 1 region was achieved through PCR, employing primer sets ITS1F (5′-CTTGG TCATTTAGAGGAAGTAA-3′) and ITS2R (5′-GCTGCGTTCTTCATCGATGC-3′). Comprising the PCR mixture were 4 μL of 5 × FastPfu buffer, 2 μL of 2.5 mM dNTPs, and 0.8 μL of each mixture. PCR amplification was conducted under these cycling parameters: 3 min of denaturation at 95 °C, 30 s of denaturation at 95 °C, 30 s of annealing at 55 °C, 45 s of extension at 72 °C, followed by a 10-min single extension at 72 °C, cumulating in 27 cycles, culminating in the reaction's completion at 4 °C. Each specimen was subjected to amplification on three separate occasions. PCR outcomes were derived from 2 % agarose gels, cleansed with the AxyPrep DNA Gel Extraction Kit (Axygen Biosciences, Union City, CA, USA) as per the guidelines provided by the manufacturer, and examined using a QuantusTM fluorometer (Promega, Madison, WI, USA).

Equal quantities of the refined amplicons were merged and analyzed through paired-end sequencing on the Illumina MiSeq PE300 system (Illumina, San Diego, CA, USA), employing the conventional techniques supplied by Majorbio Bio-Pharm Technology Co. Limited, Shanghai, China.

### 2.7 Data processing and statistical analysis

Unless stated otherwise, all experiments were conducted in triplicate. Statistical evaluation of the outcomes was conducted using version 22.0 of the SPSS software for Windows (SPSS Inc., Chicago, IL, USA). The experimental data were obtained by conducting three parallel experiments and the results were presented as the mean value plus or minus the standard deviation. The averages of treatments underwent a statistical analysis via Duncan's test, establishing statistical significance at the *p* < 0.05 threshold.

Using RDP Classifier version 2.2 (Zhang et al., 2023), the taxonomic categorization of each OTU sample sequence was scrutinized, applying a confidence level of 0.7. The microbiota was analyzed using the Majorbio cloud platform, available at https://cloud.majorbio.com. Alpha diversity was employed to assess species diversity in the samples. Beta diversity was assessed using QIIME to compare species complexity between samples, using both weighted and unweighted UniFrac methods.

## 3 Results and discussion

### 3.1 Texture profile analysis and color changes

The color changes of different samples were analyzed using a colorimeter, with L* values indicating the degree of lightness or darkness, a* values indicating the degree of redness or greenness, and b* values indicating the degree of yellow and blue (Li et al., 2020). The color variations of the low-salt shrimp paste are displayed in Table 1. Throughout the fermentation process, there was a notable decrease in the brightness L* value of three varieties of low-salt shrimp paste. In contrast, there was a significant rise in both the redness a* and yellowness b* values. The color change is related to the change of the content of the Maillard reaction and astaxanthin, products of the Maillard reaction will make the shrimp paste appear brown; the pink color of the low-salt shrimp paste is affected by the content of astaxanthin, which is concentrated in the early stage of fermentation due to the drying and salting dehydration (Pongsetkul et al., 2017). In the course of the fermentation process, the L* and b* values in group F were found to be significantly elevated compared to those in the other experimental groups. This may be related to the addition of glutinous rice flour, which was shown to improve the color of stabilized leachate (Nur & Omar, 2017). However, astaxanthin is chemically unstable and can be decomposed under the influence of external conditions such as light and oxygen (Yao et al., 2023), which can lead to the color of the shrimp paste becoming darker as fermentation duration extends, the darker the color, the more astaxanthin is broken down. During the fermentation process, the L* value of Group F was higher than that of Group P, and Group Y was the lowest. These results indicated that the addition of a carbon source effectively reduced the decomposition of astaxanthin, thereby enhancing the nutritional value of low-salt shrimp paste.

**Table 1:** Changes of texture and color parameters during fermentation of low-salt shrimp paste.

		0d	3d	6d	9d	12d
Hardness (N)	Y	60.99 ± 31.47^Bab^	35.88 ± 4.31^ABb^	73.71 ± 15.09^ABa^	52.40 ± 1.61^Aab^	56.46 ± 10.09^Bab^
	P	75.99 ± 32.07^ABab^	29.81 ± 9.87^Bc^	90.94 ± 8.99^Aa^	59.64 ± 6.37^Ac^	65.16 ± 7.96^Abc^
	F	135.99 ± 30.18^Aa^	55.42 ± 15.97^Ab^	51.09 ± 6.99^Bb^	54.11 ± 4.71^Bb^	64.63 ± 0.11^Ab^
Springiness (mm)	Y	0.65 ± 0.05^Ab^	0.84 ± 0.08^Aab^	0.83 ± 0.15^Aab^	0.89 ± 0.01^Aab^	0.82 ± 0.17^Aab^
	P	0.66 ± 0.08^Ab^	0.88 ± 0.01^Aa^	0.73 ± 0.13^Ab^	0.93 ± 0.06^Aa^	0.87 ± 0.04^Aa^
	F	0.61 ± 0.02^Ab^	0.80 ± 0.14^Aa^	0.82 ± 0.06^Aa^	0.83 ± 0.06^Aa^	0.83 ± 0.11^Aa^
Chewiness (N)	Y	24.17 ± 4.33^Bab^	12.53 ± 1.03^Bb^	29.40 ± 4.87^Aa^	27.60 ± 3.03^Aa^	22.34 ± 9.63^Aab^
	P	26.62 ± 10.26^Ba^	11.31 ± 1.48^Bb^	27.52 ± 9.12^Aa^	12.71 ± 3.25^Cb^	23.12 ± 1.60^Aab^
	F	53.12 ± 6.97^Aa^	17.68 ± 3.19^Ab^	19.24 ± 5.82^Ab^	20.17 ± 1.39^Bb^	25.41 ± 2.77^Ab^
L*	Y	56.75 ± 0.79^Ba^	54.99 ± 0.36^Ba^	51.91 ± 2.69^Ca^	49.14 ± 3.09^Ca^	47.82 ± 14.47^Aa^
	P	57.43 ± 0.93^Ba^	56.50 ± 1.75^Ba^	56.32 ± 2.52^Ba^	55.50 ± 0.48^Ba^	55.29 ± 0.62^Aa^
	F	64.82 ± 0.28^Ac^	62.78 ± 1.24^Aa^	61.05 ± 1.08^Ac^	60.32 ± 1.38^Ab^	60.15 ± 1.79^Abc^
a*	Y	11.34 ± 1.11^Ab^	14.98 ± 2.31^Ba^	15.90 ± 1.49^Aa^	16.30 ± 0.98^Aa^	16.40 ± 2.00^Aa^
	P	12.18 ± 1.53^Ac^	18.95 ± 1.52^Aa^	14.52 ± 1.69^Abc^	16.37 ± 1.62^Aab^	17.00 ± 0.33^Aab^
	F	10.65 ± 0.70^Ad^	18.71 ± 2.14^Aa^	13.37 ± 1.83^Ac^	15.40 ± 0.63^Abc^	16.64 ± 0.69^Aab^
b*	Y	17.35 ± 1.08^ABb^	19.92 ± 2.33^Aa^	17.65 ± 0.72^Ab^	17.37 ± 1.06^Bb^	18.20 ± 0.79^Bab^
	P	15.56 ± 1.86^Bb^	19.85 ± 3.44^Aa^	15.62 ± 1.31^Ab^	16.57 ± 0.66^Bab^	17.91 ± 2.67^Bab^
	F	18.26 ± 1.20^Abc^	22.26 ± 1.63^Aa^	16.77 ± 2.00^Ac^	20.07 ± 0.68^Aab^	22.00 ± 0.41^Aa^

*Note*: The data is presented as average values ± SD (*n* = 3). Averages featuring varying lowercase letters within the same row show a notable difference (*p* < 0.05), and different uppercase letters in the same column for the same parameter indicate a significant difference (*p* < 0.05).

TPA was employed to assess the textural properties of shrimp paste. Changes in pH value water content, and microbial metabolism can lead to changes in the texture of low-salt shrimp paste (Chen et al., 2016). As shown in Table 1, the hardness and chewiness of the three groups' samples decreased and springiness increased at 12 d of fermentation compared to the initial fermentation. The observed alterations can be related to the proliferation and metabolic activity of bacteria in the shrimp paste samples. The process involves releasing various extracellular enzymes that decompose myosin and actin proteins, leading to the breakdown of connective tissues and eventually making the shrimp paste softer (Peng et al., 2023). The decrease in chewiness may be related to the activity of endogenous proteases leading to hydrolysis of tissue myofibrillar proteases (Xiao et al., 2022). The quality of muscle is determined by a combination of physical properties, the higher the value of hardness, springiness, and chewiness, the better the muscle quality (Shi et al., 2024). During the fermentation process, the hardness and chewiness in group F were significantly higher than those of the samples in group Y, this may be due to the addition of glutinous rice flour improving the texture of the shrimp paste. Liu et al. (2018) found that glutinous rice flour improved the expansion, homogeneity, hardness, crunchiness, and color of imitation cheese. The low-salt shrimp paste in group P showed higher hardness and elasticity compared with those in group Y, glucose was responsible for the hardness and crispness of the pectin-glucose system, and the addition of glucose increases the strength and elasticity of the gel (Feng et al., 2023; Lopez-Sanchez et al., 2022). At the 12th d of fermentation, the hardness of shrimp paste with added glucose and glutinous rice flour was significantly higher than the control group, with slightly higher springiness and chewiness than the control group.

### 3.2 Physicochemical properties of low-salt shrimp paste at different stages of fermentation

**Fig. 1:**
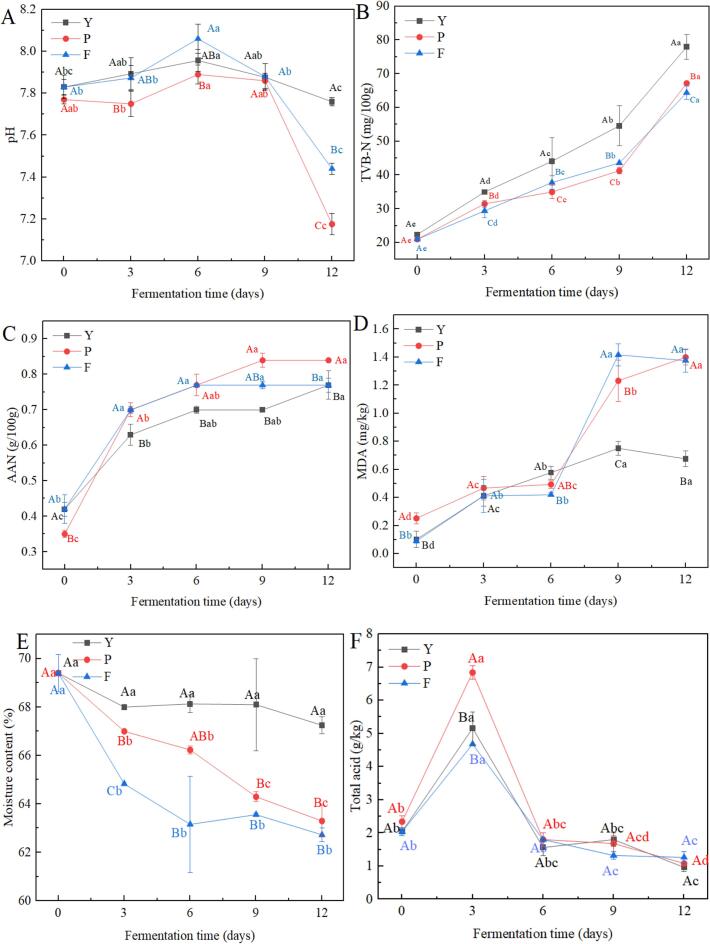
Changes in pH value(A), TVB-N (B), AAN (C), MDA (D), Moisture content (E), and total acid (F) during fermentation of low-salt shrimp paste. Different lowercase letters in the same color for the same point lot indicate significant differences (*p* < 0.05), and different capital letters for the same fermentation time indicate significant differences (*p* < 0.05).

The pH value serves as a critical safety parameter for fermented products (Li et al., 2022). As shown in Fig. 1A, the pH value in group Y increased from 7.83 ± 0.03 to 7.96 ± 0.05 during 0–6 d of fermentation and decreased to 7.76 ± 0.02 on day 12 of fermentation. During the fermentation of the low-salt shrimp paste in group P, the pH value increased from 7.77 ± 0.02 to 7.89 ± 0.05 and then decreased to 7.17 ± 0.05. The pH value of group F samples first increased from 7.83 ± 0.06 to 8.06 ± 0.07 and then decreased to 7.44 ± 0.03. In each of the three groups, the pH value tended to rise initially before falling. The pH value rises during the pre-fermentation period, this phenomenon may be caused by the low salt conditions, most of the microbial activity is not inhibited, protein excessive hydrolysis leading to the production of BAs or other basic substances, and thus increasing pH value (Che et al., 2021). The decrease in pH value may be due to the continuous decomposition of organic acids during the fermentation process (Surya et al., 2023) and a low-salt environment is conducive to the growth and metabolism of lactic acid bacteria, thereby accelerating the production of lactic acid. During the fermentation process, the pH value of group P was always lower than that of group Y. This is because glucose is oxidized to gluconic acid by the enzyme glucose oxidase, which lowers the pH value of the low-salt shrimp paste. Additionally, microorganisms can produce lactic acid through the fermentation of glucose (Christian & Peter, 2006). The pH value in group F was higher than that of group Y in 6 d, probably because the increase in glutinous rice flour caused a sharp increase in *Alkalibacterium* during the fermentation of the shrimp paste (Group F in Fig. 5 showed a significant increase in *Alkalibacterium* on day 6 of fermentation), which led to the appearance of an increase in pH value.

TVB-N is commonly utilized as a biomarker to assess the spoilage of fermented food products (Bekhit et al., 2021). Fig. 1B illustrates a notable rise in TVB-N levels in each of the three low-salt shrimp paste groups correlating with the duration of fermentation (*p* < 0.05). The TVB-N value of low-salt shrimp paste increased from 22.4 ± 0.02 mg/100 g to 78 ± 3.66 mg/100 g in group Y and from 21 ± 0.05 mg/100 g to 67.2 mg/100 g and 64.4 ± 1.98 mg/100 g in groups P and F respectively. The increases resulted from the expedited deterioration of muscle proteins due to the activity of internal enzymes (Shiekh et al., 2021). Throughout the fermentation period, the TVB-N values in Groups P and F were found to be lower than those in Group Y. This suggests that the inclusion of glucose and glutinous rice flour successfully hindered the rise of TVB-N in the low-salt shrimp paste samples.

AAN serves as a significant biomarker of the fermentation quality and extent of fermentation in food, the higher the content of AAN, the higher the degree of fermentation, and the better the flavor (Ruan et al., 2022). As shown in Fig. 1C, the AAN content of the three groups of low-salt shrimp paste increased rapidly from 0 to 3 days of fermentation, samples of group Y increased from 0.42 ± 0.02 g/100 g to 0.63 ± 0.03 g/100 g, shrimp paste of group P increased from 0.35 ± 0.01 g/100 g to 0.70 ± 0.02 g/100 g, and shrimp paste of group F increased from 0.42 ± 0.04 g/100 g to 0.70 ± 0.01 g/100 g. At 3–12 d of fermentation, the AAN content of the three groups of shrimp paste increased slowly compared to that at the beginning of fermentation, which is similar to the results of Lee's study (Lee, Jung and Jeon, 2014a, Lee, Jung and Jeon, 2014b) of changes in AAN in the effect of different temperatures on saeu-jeot fermentation. In the initial phase of fermentation, the swift rise in AAN levels could stem from microorganisms releasing diverse enzymes that break down proteins in the shrimp paste into amino acids and small molecule peptides, among others. However, during the fermentation's middle and later stages, this could be attributed to the slow dissolution and absorption of salt, heightened salt content in the shrimp paste, and reduced activity of internal enzymes, resulting in a deceleration of protein decomposition (Lee et al., 2016). It is worth noting that the AAN content of low-salt shrimp paste in both groups P and F was higher than that of group Y during fermentation, possibly because glucose increases the total enzyme activity of the protease, glucose mainly promotes the production and activity of proteases by providing energy, regulating gene expression, and altering environmental conditions during fermentation, leading to an increase in free amino acids (Canan, 2022), rice proteins may be present in the glutinous rice flour itself, which breaks down into AAN (Pereira et al., 2019). Indicating that the addition of glucose and glutinous rice flour was beneficial in increasing the flavor of low-salt shrimp paste.

Animal lipids are prone to react to produce aldehydes such as MDA, which is one of the byproducts of lipid oxidation, reflecting the lipid oxidation status of the food, and high levels of which can negatively affect the structure, flavor, and color of the food (Li et al., 2022). MDA is frequently employed to gauge the degree of lipid oxidation present in fermentation byproducts (Wang et al., 2022). The MDA values of low-salt shrimp paste during fermentation are shown in Fig. 1D. The value increased from 0.11 ± 0.06 mg/kg to 0.68 ± 0.06 mg/kg in group Y and increased from 0.25 ± 0.04 mg/kg to 1.40 ± 0.05 mg/kg in group P and from 0.09 ± 0.01 mg/kg to 1.38 ± 0.08 mg/kg in group F. The change may be caused by the oxidation of fat during fermentation (Takeungwongtrakul et al., 2012). During the fermentation days 0–3, the MDA content in the P group was higher than in the Y group, while the MDA values in the F group were similar to those in the Y group. During the fermentation days 3–6, the MDA values in the P and F groups of shrimp paste were slightly lower than in the Y group. The MDA values of groups P and F were significantly higher than that of group Y at the late fermentation stage of 6–12 d, this may be due to changes in the microbial community in the shrimp paste after the addition of glucose and glutinous rice flour. *Staphylococcus* and unclassifiedf_Bacillaceae showed a significant positive correlation with MDA, suggesting that these microorganisms might have promoted lipid peroxidation reactions during the fermentation process. On the other hand, it may be because the added glutinous rice flour itself contains lipids that will be utilized by the microorganisms in the fermentation environment (Siriamornpun et al., 2016). The addition of glucose in Group P shrimp sauces may have accelerated lipid oxidation, with glucose potentially enhancing MDA by aiding in the generation of trace amounts of 4-HNE, a byproduct of lipid peroxidation (Cohen et al., 2011).

The moisture content of low-salt shrimp paste during fermentation is shown in Fig. 1E. The Chinese aquaculture industry standard stipulates that the moisture content in shrimp paste should be ≤60 %. However, the moisture content measured in the samples is higher than the standard. The possible reason for this phenomenon is that the low-salt shrimp paste contains a lower amount of salt compared to traditional shrimp paste, which results in a higher moisture content. During the fermentation process, the moisture content of all three groups of shrimp paste showed a gradually decreasing trend. Notably, the moisture content of the P and F groups was significantly lower than that of the Y groups. This is because glucose has strong hygroscopic properties (i.e., it can absorb and retain moisture (Zhu et al., 2024). After glucose was added to the P group, it absorbed moisture from the surrounding environment and combined with the water, thereby reducing the free water content in the shrimp paste. The addition of glutinous rice flour in the F group reduced the water loss during fermentation through mechanisms such as water absorption, gelation, and water binding (Lai, 2001), resulting in a decrease in moisture content during the fermentation process.

The total acids of low-salt shrimp paste during fermentation are shown in Fig. 1F. The trend in the total acid content of the three groups of shrimp paste is similar. The total acid content increased during the first three days of fermentation, after which it gradually decreased and leveled off. This trend is consistent with the findings of Xu et al. (2008) in their study on the rapid fermentation of fish sauce. Moreover, Montel et al. (1996) suggested that lactic acid reaches its highest concentration during the mid-stage of fermentation, and then declines. On the third day of fermentation, the total acid content of the P group shrimp paste was significantly higher than that of the Y and F groups. This is because the addition of glucose provides an additional carbon source, promoting microbial fermentation and metabolism, which produces more organic acids, leading to an increase in the total acid content during the fermentation of shrimp paste (Christian & Peter, 2006). The total acid content of the F group did not differ significantly from that of the Y group, as glutinous rice flour mainly consists of starches, which are hard converted rapidly into acidic substances and thus have a smaller direct effect on the total acid content.

### 3.3 Amino acid analysis and histamine analysis of low-salt shrimp paste at different stages of fermentation

Free amino acids (FAAs) not only possess their unique flavor potential but also serve as the forerunners to numerous volatile flavor compounds. As indicated in Table 2, a total of 15 FAAs were identified in all samples of low-salt shrimp paste. The FAAs were classified into three groups according to taste: umami taste (Aspartic acid, Glutamic acid, Glycine, and Alanine), sweetness (Threonine, Lysine, Serine, and Proline), bitterness (Valine, Histidine, Isoleucine, Leucine, Phenylalanine, Methionine, and Arginine) (Shang et al., 2022).

**Table 2:** Changes of free amino acid content in shrimp paste during fermentation.

Amino acids content (μg/g)	U	Y - 6	Y - 12	P - 6	P - 12	F - 6	F - 12
Umami amino acid	3.11 ± 0.26^d^	3.92 ± 0.01^b^	2.96 ± 0.01^d^	5.19 ± 0.02^a^	3.43 ± 0.01^c^	4.99 ± 0.08^a^	3.57 ± 0.01^c^
Aspartic acid	0.02 ± 0.01^g^	0.39 ± 0.01^f^	0.74 ± 0.01^e^	2.06 ± 0.02^a^	0.96 ± 0.01^d^	1.79 ± 0.06^b^	1.04 ± 0.01^c^
Glutamic acid	0.60 ± 0.04^e^	0.64 ± 0.01^e^	1.34 ± 0.01^c^	1.66 ± 0.01^b^	0.64 ± 0.03^e^	1.76 ± 0.02^a^	1.09 ± 0.0^d^
Glycine	1.41 ± 0.16^a^	1.29 ± 0.01^b^	0.05 ± 0.02^d^	0.32 ± 0.01^bc^	0.49 ± 0.01^b^	0.29 ± 0.01^c^	0.38 ± 0.01^bc^
Alanine	1.08 ± 0.08^cd^	1.61 ± 0.02^a^	0.84 ± 0.01	1.15 ± 0.01^cd^	1.35 ± 0.01^b^	1.16 ± 0.01^c^	1.07 ± 0.02^d^
Sweet amino acid	1.62 ± 0.03^e^	6.08 ± 0.09^c^	3.10 ± 0.03^d^	8.10 ± 0.01^a^	3.10 ± 0.02^d^	7.51 ± 0.14^b^	3.12 ± 0.01^d^
Threonine	0.22 ± 0.01^e^	1.97 ± 0.01^c^	0.06 ± 0.01^f^	4.84 ± 0.01^a^	0.34 ± 0.01^d^	4.65 ± 0.05^b^	0.32 ± 0.01d
Lysine	1.07 ± 0.02^e^	2.80 ± 0.01^a^	2.62 ± 0.01^b^	2.31 ± 0.04^d^	2.42 ± 0.01^c^	2.62 ± 0.03^d^	2.45 ± 0.01^c^
Serine	0.28 ± 0.01^f^	1.04 ± 0.01^a^	0.42 ± 0.01^d^	0.95 ± 0.02^b^	0.33 ± 0.02^e^	0.60 ± 0.05^c^	0.34 ± 0.01^e^
Proline	0.06 ± 0.02^b^	0.27 ± 0.07^a^	ND	ND	ND	ND	ND
Bitter amino acid	6.51 ± 0.07^g^	11.60 ± 0.03^f^	16.93 ± 0.02^c^	17.53 ± 0.02^b^	16.27 ± 0.01^e^	16.50 ± 0.23^d^	19.73 ± 0.02^a^
Valine	0.58 ± 0.01^e^	0.60 ± 0.01^e^	0.51 ± 0.01^f^	1.98 ± 0.01^a^	1.38 ± 0.01^c^	1.78 ± 0.04^b^	1.06 ± 0.01^d^
Histidine	0.29 ± 0.01^f^	0.74 ± 0.01^e^	1.06 ± 0.02^c^	1.11 ± 0.01^b^	1.14 ± 0.01^a^	0.89 ± 0.02^d^	1.11 ± 0.01^b^
Isoleucine	0.48 ± 0.01^f^	1.80 ± 0.03^d^	1.56 ± 0.01^e^	3.51 ± 0.01^a^	2.32 ± 0.01^c^	3.34 ± 0.04^b^	2.34 ± 0.01^c^
Leucine	0.94 ± 0.01^d^	2.93 ± 0.01^b^	2.60 ± 0.01^c^	ND	ND	ND	3.89 ± 0.03^a^
Phenylalanine	0.60 ± 0.01^g^	1.98 ± 0.01^f^	2.91 ± 0.01^a^	2.54 ± 0.01^d^	2.78 ± 0.01^b^	2.45 ± 0.03^e^	2.69 ± 0.01^c^
Methionine	0.43 ± 0.01^g^	1.31 ± 0.01^f^	1.78 ± 0.04^a^	1.59 ± 0.01^d^	1.72 ± 0.01^c^	1.52 ± 0.02^e^	1.74 ± 0.01^b^
Arginine	3.19 ± 0.07^d^	2.23 ± 0.04^e^	6.50 ± 0.01^c^	6.79 ± 0.02^b^	6.94 ± 0.01^a^	6.53 ± 0.08^c^	6.89 ± 0.01^ab^
Total free amino	11.25 ± 0.29^f^	21.60 ± 0.13^e^	23.00 ± 0.01^d^	30.82 ± 0.03^a^	22.80 ± 0.02^d^	29.01 ± 0.45^b^	26.42 ± 0.01^c^
acid (TFAA)							

*Note*: U for fresh unfermented raw material. Y, P, and F represent different fermentation groups of low-salt shrimp paste, numbers indicate days of fermentation. Lowercase letters in the same row indicate significant differences (*p* < 0.05).

As illustrated in Table 2, the total free amino acid content (TFAA) in group Y (the control group) exhibited a consistent upward trend throughout the fermentation process. Conversely, groups P and F demonstrated an initial increase in TFAA followed by a subsequent decline. TFAA of fresh unfermented raw material was 11.25 ± 0.29 μg/g, low-salt shrimp in group Y increased to 23.00 ± 0.01 μg/g at day 12 of fermentation, samples from group P first increased to 30.82 ± 0.03 μg/g and then decreased to 22.80 ± 0.02 μg/g, and group F first increased to 29.01 ± 0.45 μg/g and then decreased to 26.42 ± 0.01 μg/g. Hou et al. (2011) found that the amino acid content of dried scallop-flavored sauce increased during the fermentation process, and Gao et al. (2023) showed the same trend of changes in low-salt shrimp paste in groups P and F. Throughout the fermentation process, alterations in FAAs were facilitated by internal enzymes and microorganisms (Wang et al., 2020). During the initial phases of fermentation, microorganisms actively decompose proteins, resulting in an elevated concentration of amino acids. Group P had the highest TFAA content of 30.82 ± 0.03 μg/g on day 6 of fermentation, probably due to the high abundance of *Staphylococcus* of 20.72 %, *Staphylococcus* demonstrated protease functions, capable of breaking down proteins into peptides and FAAs (Yue et al., 2016). During the fermentation days 6–12, the TFAA content in groups P and F significantly (*p* < 0.05) decreased, this may be because the addition of glucose or glutinous rice flour changed the microbial communities and also caused the microorganisms to preferentially use carbon sources rather than rely on nitrogen sources (such as amino acids from protein hydrolysis), which could lead to a decrease in the free amino acid content in the shrimp paste. The increase in free amino acid content in the Y group during days 6–12 may be due to the absence of external carbon source interference during its fermentation process, allowing microbial metabolic activity to more directly promote the synthesis and accumulation of amino acids.

The composition and content of FAAs of umami and sweet flavors are related to the degree of freshness of the product, which can enhance the characteristic flavor, sweetness, taste persistence, and complexity of the food (Guo et al., 2023). As shown in Table 2, the umami amino acid content of fresh unfermented raw material was 3.11 ± 0.26 μg/g, which increased to 3.92 ± 0.01 μg/g and then decreased to 2.96 ± 0.01 μg/g in group Y shrimp paste, increased to 5.19 ± 0.02 μg/g and then decreased to 3.43 ± 0.01 μg/g in group P, increased to 4.99 ± 0.08 μg/g and then decreased to 3.57 ± 0.01 μg/g in group F. The umami amino acid content of fresh unfermented raw material was 3.11 ± 0.26 μg/g in groups Y, P, and F, which showed an increasing and then decreasing trend. The sweet amino acid content of fresh unfermented raw material was 1.62 ± 0.03 μg/g, and the shrimp paste of group Y first increased to 6.08 ± 0.09 μg/g and then decreased to 3.10 ± 0.03 μg/g, the P group first increased to 8.10 ± 0.01 μg/g and then decreased to 3.10 ± 0.02 μg/g, group F first increased to 7.51 ± 0.14 μg/g and then decreased to 3.12 ± 0.01 μg/g. The trends of umami and sweet amino acids in groups P and F were similar to group Y. Nonetheless, during fermentation, the levels of umami and sweet amino acids in groups P and F notably exceeded those in group Y, with the flavor becoming more distinct. This may be due to the addition of glucose and glutinous rice flour to promote FAA production (Teng et al., 2023). The increase in the amino acid content of shrimp paste in group F may be due to the presence of proteins in dry glutinous rice flour (Eakkanaluksamee & Anuntagool, 2020). Duan et al. (2023) showed that the specific kind and quantity of amino acids had a significant effect on the scent and taste of fermentation products. This result suggests that the addition of glucose and glutinous rice flour may enhance the richness of aroma and improve the taste of low-salt shrimp paste.

The content of bitter amino acids in fresh unfermented raw material was 6.51 μg/g, during the fermentation process of shrimp paste, it increased to 16.93 μg/g in the Y group and 19.73 μg/g in the F group, for the P group, it increased to 17.53 μg/g then decreased to 16.27 μg/g. Bitter amino acids are generally considered to have an unpleasant bitter taste, but the interaction between bitter amino acids and umami substances could mask the bitterness, and the salt taste of fermented products could offset the bitterness of bitter amino acids to some extent (Harmon et al., 2024; Li et al., 2024). Therefore, the increase of bitter amino acids had no significant effect on the overall flavor, and the accumulation of free amino acids increased the flavor richness of fermented shrimp paste.

**Fig. 2:**
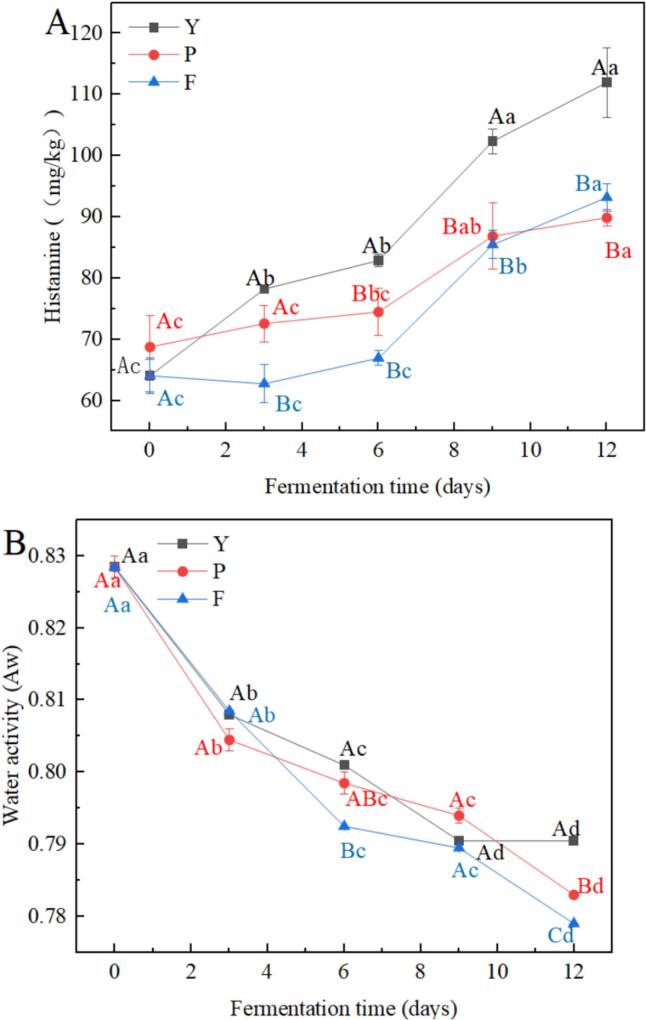
Changes in histamine (A) and water activity (B) during fermentation of low-salt shrimp paste. Different lowercase letters in the same color for the same point lot indicate significant differences (*p* < 0.05), and different capital letters for the same fermentation time indicate significant differences (*p* < 0.05).

Among the common biogenic amines in fermented foods, histamine and tyramine (TRY) are considered to be the most dangerous substances. The guidance level stated by the U.S. Food and Drug Administration (FDA) for histamine is 50 mg/kg, and the maximum established by the European Food Safety Authority (EFSA) in fresh fish is 200 mg/kg (Anal et al., 2020). The highest histamine content in the three groups of shrimp paste during fermentation was 111.96 ± 5.70 mg/kg, which complies with the ESFA standards. As shown in Fig. 2A, the histamine produced during the fermentation of the three groups of shrimp paste showed a gradual increasing trend. Histamine in group Y increased from 64.05 ± 2.97 mg/kg to 111.96 ± 5.70 mg/kg, increased from 68.74 ± 5.09 mg/kg to 89.86 ± 1.36 mg/kg in group P, and from 64.05 ± 2.63 mg/kg to 96.16 ± 2.23 mg/kg in group F. During 6–12 d of fermentation, histamine produced by group P and group F was significantly lower than that of the control group, suggesting that the addition of glucose and glutinous rice flour inhibited the production of histamine. Jae-Hyung and Hwang (2008) found that additives such as glucose also significantly reduced the production of biogenic amines in the simulated system and reduced histamine by 43.2 % in fermented anchovies. This may be due to the Maillard reaction between glucose and histamine (Jiang et al., 2017), which consumes histamine, leading to a reduction in histamine content in group P. The reduction of histamine in group F shrimp paste may be attributed to the addition of glutinous rice flour, which altered the pH value, nutrient composition, and colony taxa of fermented shrimp paste and further inhibited the growth and reproduction of histamine-producing microorganisms, thereby reducing the histamine in the shrimp sauce.

As shown in Fig. 2B, during the fermentation of the shrimp paste, the water activity of the Y group initially decreases and then stabilizes, while the water activity of the P and F groups continuously decreases. This may be due to the reduction of water activity through osmotic pressure in a low-salt environment, and the evaporation effect directly leading to the decrease in water activity (Cedeno et al., 2022). On the 6th and 12th days of fermentation, the water activity of the Y group was significantly higher than that of the P group, and the P group was significantly higher than that of the F group (*p* < 0.05). This may be due to the fact that the addition of glucose and glutinous rice flour enhances microbial metabolic activity, which in turn accelerates the absorption and conversion of moisture. The glucose added in the P group has hygroscopic properties (Zhu et al., 2024), allowing it to absorb moisture, which prevents the water from easily moving or evaporating freely, thereby reducing the water activity. The glutinous rice flour added in Group F contains a large amount of starch, which can absorb water during the fermentation process and combine with it to form a gel-like structure (Tao et al., 2024). This causes the moisture to be trapped between the starch molecules, reducing the water activity.

### 3.4 Microbiome analysis using high-throughput techniques

#### 3.4.1 Alpha diversity of the bacterial community

Alpha diversity refers to the diversity within a particular region or ecosystem. Table 3 demonstrates that the coverage index for the three shrimp paste samples exceeded 0.99, this suggests the sequencing results achieved a highly dependable quality of data. Species diversity can be depicted by the Shannon and Simpson indices (Mi et al., 2022). The higher the Shannon index, the more diverse the microbiome. Conversely, the Simpson index is the inverse (Zhao et al., 2022). With the increase of fermentation time, the microbial diversity in group Y showed a decreasing and then increasing trend, while the species diversity of shrimp paste communities in groups P and F showed a gradually decreasing trend. It was shown that the addition of glucose and glutinous rice flour had an inhibitory effect on the microbial community in low-salt shrimp paste, which altered the structure of the microbial community during the fermentation of shrimp paste and reduced the diversity of microbial species by the external carbon source.

**Table 3:** Alpha diversity index of bacterial community in low-salt shrimp paste during fermentation.

Samples	OTU	Shannon	Simpson	ACE	Chao1	Coverage
U	694 ± 13^a^	3.88 ± 0.05^a^	0.08 ± 0.01^d^	725.18 ± 35.65^a^	733.86 ± 5.20^a^	0.99
Y-6	197 ± 3^c^	1.77 ± 0.07^e^	0.31 ± 0.07^a^	226.60 ± 65.90^b^	209.68 ± 47.51^c^	0.99
Y-12	83 ± 5^d^	2.07 ± 0.11^c^	0.21 ± 0.02^b^	86.15 ± 6.51^c^	86.06 ± 7.10^d^	0.99
P-6	218 ± 9^b^	2.31 ± 0.08^b^	0.15 ± 0.01^c^	279.60 ± 39.81^b^	273.02 ± 39.19^b^	0.99
P-12	72 ± 2^d^	1.91 ± 0.05^d^	0.24 ± 0.01^b^	77.97 ± 5.03^c^	79.17 ± 3.62^d^	0.99
F-6	199 ± 8^c^	2.31 ± 0.03^b^	0.15 ± 0.01^c^	251.51 ± 7.08^b^	245.36 ± 8.43^bc^	0.99
F-12	81 ± 4^d^	2.29 ± 0.01^b^	0.17 ± 0.01^c^	88.64 ± 5.78^c^	92.17 ± 11.50^d^	0.99

*Note*: U for fresh unfermented raw material. Y, P, and F represent different fermentation groups of low-salt shrimp paste, numbers indicate days of fermentation. All values are expressed as the mean (n = 3) ± standard deviation. Different letters in the same column mean significant difference at *p* < 0.05.

Larger values of the ACE index indicate a more speciose environment with a more even distribution of species, and larger values of the Chao1 index indicate a greater number of species in the sample. Wang et al. (2022) found that the microbial community richness and diversity of low-salt sour meat are higher than that of traditional sour meat. The ACE and Chao1 indices showed higher species richness and abundance of low-salt shrimp paste in groups P and F than in group Y, with the highest species richness in Group F. It suggests that the addition of glucose and glutinous rice flour increased the species richness in the shrimp paste, and the addition of glutinous rice flour had a more pronounced effect. The species richness of the three groups of low-salt shrimp paste showed a gradual decrease with longer fermentation time.

**Fig. 3:**
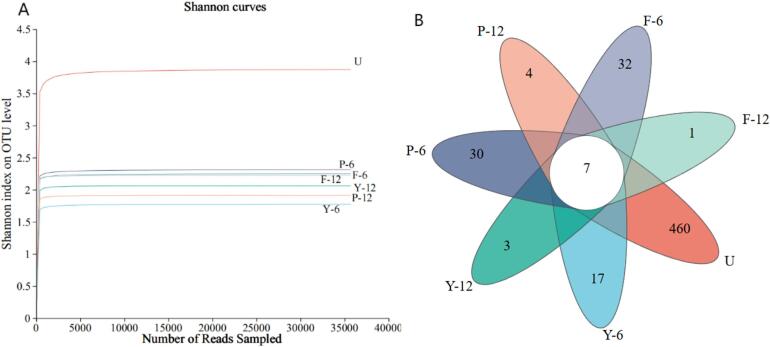
Microbial Shannon curves of low-salt shrimp paste during fermentation (A) and Venn analysis of OTU distribution of low-salt shrimp paste (B). U for fresh unfermented raw material. Y, P, and F represent different fermentation groups of low-salt shrimp paste, numbers indicate days of fermentation.

As shown in Fig. 3A, the information on the species of the bacterial community was more diverse when the shrimp paste was unfermented on the 0th day. With the increase of fermentation time of shrimp paste, the species information of shrimp paste flora in group Y decreased and then increased, and group P and group F showed a gradually decreasing trend. Suggests that the addition of glucose and glutinous rice flour has an inhibitory effect on the microbial community in shrimp paste. The Venn graph shows the distribution of OTUs for low-salt shrimp paste (Fig. 3B). The bacterial community unique to the fresh raw material was 460 OTUs. The three groups of shrimp paste contained 7 OTUs in common during fermentation, and on the 12th d of fermentation, the number of species unique to groups Y, P, and F was 3, 4, and 1, respectively. This aligns with the findings of the Alpha Diversity Index investigation mentioned earlier.

#### 3.4.2 Beta diversity cluster analysis

**Fig. 4:**
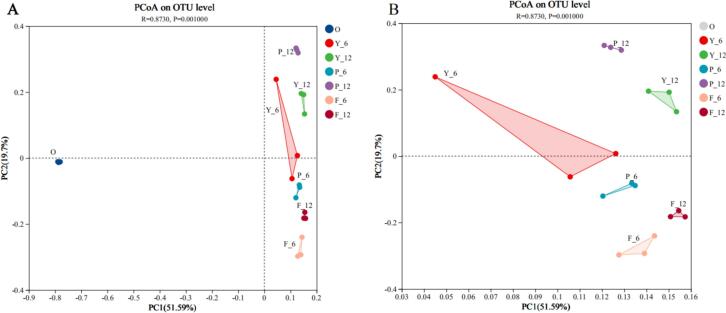
PCoA scores of bacterial communities in fermented shrimp paste under different treatments (A) and PCoA scores of three groups of shrimp paste in the middle and late stages after 0 days of removal of fermentation (B). Y, P, and F represent different fermentation groups of low-salt shrimp paste, numbers indicate days of fermentation.

A PCoA based on Bray-Curtis distances revealed differences in communities between different low-salt shrimp pastes. Dots of varying hues correspond to distinct clusters of samples, and the proximity of the dots indicates the degree of similarity in species composition between the two samples (Jia et al., 2024). As shown in Fig. 4A, all three groups of low-salt shrimp paste can be clearly distinguished from 0 d in the middle and late fermentation, which may be due to the higher activity of the strains during the pre-fermentation process resulting in a more pronounced change in the bacterial community. Fig. 4B shows that the species composition of the three groups of shrimp paste at 6 d of fermentation was more clearly distinguished from that of the same group at 12 d. On the 6th d of fermentation, the community structure of the three groups of shrimp paste could be clearly distinguished. On the 12th d of fermentation, there were differences in the bacterial colony structure in the Y, P, and F groups. It indicated that the addition of glucose and glutinous rice flour had a more significant effect on the microbiota of shrimp paste.

#### 3.4.3 Bacterial community composition

Table 4 presents the structure of microbial communities at both the phylum and family levels during the fermentation of three shrimp paste species. At the phylum level, the compositions were similar in the three low-salt shrimp pastes, with Firmicutes, Proteobacteria, Bacteroidetes, and Actinobacteria dominating all three shrimp pastes. This is the same microbial community composition of sand crab juice in the same fermentation environment as in Luo's study (Luo et al., 2021). In the preliminary stage of fermentation, Firmicutes, Proteobacteria, and Bacteroidetes were the dominant phylum, and Proteobacteria accounted for the largest proportion of 63.05 %. The relative abundance of the majority of the phyla decreased in group Y shrimp paste on day 6 of fermentation, with only Firmicutes increasing to 98.4 % and 99.9 % on 12 d. As the fermentation progressed, the proportion of Proteobacteria in groups P and F gradually decreased, and after the 6th d of fermentation, Firmicutes gradually replaced Proteobacteria as the dominant phylum. This may be because the dominant flora in shrimp paste is closely related to the microorganisms in the raw shrimp (Phewpan et al., 2020).

**Table 4:** Relative abundances of bacterial phylum and family levels at different stages of fermentation.

	Classification	U (%)	Y-6 (%)	Y-12 (%)	P-6 (%)	P-12 (%)	F-6 (%)	F-12 (%)
Phylum	Firmicutes	16.64	98.48	99.94	97.52	99.99	98.88	99.96
	Proteobacteria	63.05	0.90	0.06	1.76	0.01	0.62	0.00
	Bacteroidota	9.3	0.22	0.00	0.32	0.00	0.31	0.00
	Actinobacteriota	5.12	0.28	0.00	0.18	0.00	0.00	0.00
	Verrucomicrobiota	1.89	0.00	0.00	0.08	0.00	0.00	0.00
	others	3.95	0.12	0.00	0.14	0.00	0.19	0.04
Family	Bacillaceae	0.00	56.17	63.89	32.37	57.31	30.35	44.18
	Staphylococcaceae	0.71	35.58	32.54	50.38	37.21	38.23	29.64
	Carnobacteriaceae	0.00	3.11	0.93	13.47	4.31	27.54	23.10
	Vibrionaceae	32.61	0.13	0.00	0.25	0.00	0.06	0.00
	unclassified_c__Bacilli	0.00	0.37	2.58	0.44	1.17	1.51	3.04
	Alcaligenaceae	7.33	0.42	0.00	0.49	0.00	0.18	0.00
	Entomoplasmatales_Incertae_Sedis	7.11	0.12	0.00	0.65	0.00	0.18	0.00
	Rhodobacteraceae	6.02	0.15	0.00	0.25	0.00	0.06	0.00
	Mycoplasmataceae	6.21	0.08	0.00	0.00	0.00	0.00	0.00
	Flavobacteriaceae	4.77	0.00	0.00	0.08	0.00	0.07	0.00
	Pseudomonadaceae	3.76	0.00	0.00	0.12	0.00	0.00	0.00
	Xanthomonadaceae	3.10	0.00	0.00	0.27	0.00	0.00	0.00
	Saprospiraceae	2.49	0.10	0.00	0.19	0.00	0.00	0.00
	Moraxellaceae	2.01	0.00	0.00	0.00	0.00	0.00	0.00
	Rubritaleaceae	1.67	0.00	0.00	0.06	0.00	0.08	0.00
	Micrococcaceae	1.22	0.17	0.00	0.08	0.00	0.00	0.00
	Rhizobiaceae	1.41	0.15	0.00	0.00	0.00	0.08	0.00
	Sphingobacteriaceae	1.32	0.70	0.00	0.07	0.00	0.02	0.00
	Stappiaceae	1.27	0.00	0.00	0.00	0.00	0.00	0.00
	Beijerinckiaceae	1.19	0.00	0.00	0.09	0.00	0.08	0.00
	others	15.70	2.75	0.06	0.74	0.00	1.56	0.04

*Note:* U for fresh unfermented raw material. Y, P, and F represent different fermentation groups of low-salt shrimp paste, numbers indicate days of fermentation.

At the family level, 20 bacterial families (relative abundance >1 %) were identified in low-salt shrimp paste throughout the fermentation process, including Bacillaceae, Staphylococcaceae, Carnobacteriaceae, Vibrionaceae, unclassified_c__Bacilli, Alcaligenaceae, Entomoplasmatales_Incertae_Sedis, Rhodobacteraceae, Mycoplasmataceae, Flavobacteriaceae, Pseudomonadaceae, Xanthomonadaceae, Saprospiraceae, Moraxellaceae, Rubritaleaceae, Micrococcaceae, Rhizobiaceae, Sphingobacteriaceae, Stappiaceae and Beijerinckiaceae. While glucose, as a carbon source, might promote the growth of pathogenic bacteria in some cases, it also provides essential nutrients for probiotics (Granados-Arvizu et al., 2019). In contrast, glutinous rice flour is primarily used as a starchy substance to provide an additional source of energy and may support the growth of specific flora under certain conditions. At the beginning of fermentation, the bacterial community composition is very complex and has a rich diversity. The predominant bacterial families in fermentation day 0 (U) were Vibrionaceae (32.61 %), Alcaligenaceae (7.33 %), Entomoplasmatales_Incertae_Sedis (7.11 %), Rhodobacteraceae (6.02 %), and Mycoplasmataceae (6.21 %). In the middle and late stages of fermentation, the main bacterial families of the three groups were Bacillaceae, Staphylococcaceae, and Carnobacteriaceae, which was similar to the results of the traditional shrimp paste in the Jinzhou area (Lv et al., 2020). During the fermentation of shrimp paste in group Y, Bacillaceae increased from not detected (U) to 63.89 % (Y-12), Staphylococcaceae increased from 0.71 % (U) to 35.58 % (Y-6) and then decreased to 32.54 % (Y-12), Carnobacteriaceae increased from not detected (U) to 3.11 % (Y-6) and then decreased to 0.93 % (Y-12). During the fermentation of shrimp paste in group P, Bacillaceae increased from not detected (U) to 57.31 % (P-12), Staphylococcaceae increased from 0.71 % (U) to 50.38 % (P-6) and then decreased to 37.21 % (P-12), Carnobacteriaceae increased from not detected (U) to 13.47 % (P-6) and then decreased to 4.31 % (P-12). During the fermentation of shrimp paste in group F, Bacillaceae increased from not detected (U) to 44.18 % (F-12), Staphylococcaceae increased from 0.71 % (U) to 38.23 % (F-6) and then decreased to 29.64 % (F-12). Carnobacteriaceae increased from not detected (U) to 27.54 % (F-6) and then decreased to 23.10 % (F-12). Some of the Bacillaceae were pathogenic (Harirchi et al., 2022; Torres-Quintero et al., 2016), while the addition of glucose and glutinous rice flour reduced the relative abundance of Bacillaceae in the fermentation of shrimp paste. During the fermentation in group P, the relative abundance of Staphylococcaceae was consistently higher than that of group control and group F, which may be related to the addition of glucose.

**Fig. 5:**
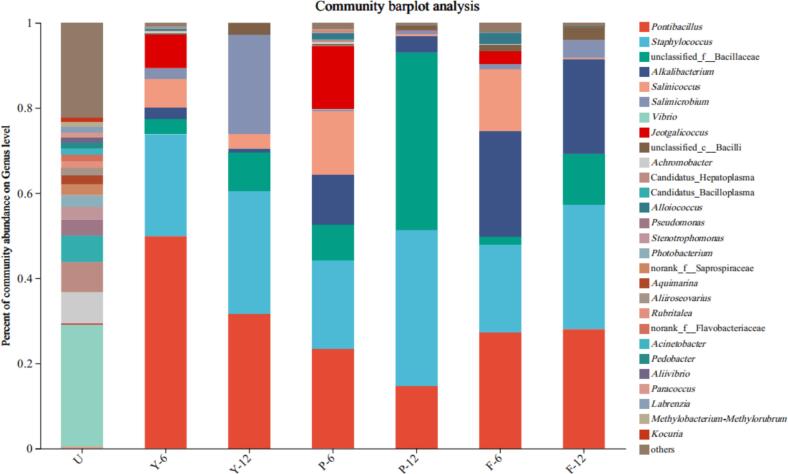
Bacterial diversity of low-salt shrimp paste at the genus level. U for fresh unfermented raw material. Y, P, and F represent different fermentation groups of low-salt shrimp paste, numbers indicate days of fermentation.

As shown in Fig. 5, at the genus level, a total of 28 major genera were identified (relative abundance >1 %), including *Pontibacillus*, *Staphylococcus*, nclassified_f__Bacillaceae, *Alkalibacterium*, *Salinicoccus*, *Salimicrobium*, *Vibrio*, *Jeotgalicoccus* and unclassified__c__Bacilli. This is similar to the traditional Thai shrimp paste fermentation study (Phewpan et al., 2020). *Salinicoccus* and *Salimicrobium* are moderately halophilic bacteria that grow in a wide pH range, with 5–10 and 24 % NaCl being the highest range (Choi et al., 2014). *Vibrio* are prevalent in the shrimp gut (Wanilada et al., 2014). At the early stage of shrimp paste fermentation (U), the composition of microbial genera was relatively complex, and the main genera were *Vibrio* (28.6 %), *Achromobacter* (7.33 %), *Pseudomonas* (7.11 %), *Candidatus_Hepatoplasma* (6.20 %) and *Pseudomonas* (3.76 %). In the fermentation process, *Pontibacillus*, *Staphylococcus*, unclassified_f__Bacillaceae, *Alkalibacterium*, *Salinicoccus*, and *Salimicrobium* became the dominant genus, which may be because salt-tolerant microorganisms are more likely to survive in a saline environment. On the 6th day of fermentation, *Pontibacillus* and *Staphylococcus* in group Y were the dominant strains. On day 12 of fermentation, *Salimicrobium* increased from 2.47 % on day 6 to 23.18 %, which was the dominant group along with *Pontibacillus* and *Staphylococcus*. This is similar to the results of Lee, Jung and Jeon, 2014a, Lee, Jung and Jeon, 2014b, where *Salimicrobium* ended up being the dominant genus in samples with 28 g/100 mL salt addition. In group P shrimp paste fermentation, *Pontibacillus*, *Staphylococcus*, *Alkalibacterium*, *Salinicoccus*, and *Jeotgalicoccus* were more predominant at day 6, while at day 12, *Pontibacillus*, *Staphylococcus* with the unclassified_f__Bacillaceae were the dominant genera. The relative abundance of unclassified_f__Bacillaceae increased to 40.71 % from 8.33 % on day 6. This may be due to the addition of glucose to the shrimp paste at the later stage of fermentation. The top four genera accounting for relative abundance on day 6 in group F were *Pontibacillus*, *Staphylococcus*, *Alkalibacterium*, and *Salinococcus*. The dominant genera on day 12 were *Pontibacillus*, *Staphylococcus*, and *Alkalibacterium*. *Alkalibacterium* was also detected in Thai traditional shrimp paste during fermentation, which is similar to our results. *Alkalibacterium* as a whole showed an increasing and then decreasing trend, which is the same as the trend from stage 2 to stage 8 in the study of Dai et al. (2018). Wang et al. (2022) found that *Staphylococcus* was also present throughout the fermentation process and was able to degrade branched-chain amino acids and fatty acids, enhancing the color and flavor of fermented products, this is consistent with our results. On the 12th day of fermentation, the relative abundance of *Staphylococcus* in group P (36.50 %) was higher than in group F (29.28 %), which in turn was higher than in group Y (26.83 %). *Staphylococcus* was also detected in fish sauce (Liu et al., 2017). It was found that *Staphylococcus carnosus* M43 could reduce biogenic amines during the fermentation process of soybean paste (Zhao et al., 2020). This may be one of the reasons why the addition of glucose and glutinous rice flour reduces the biogenic amines.

**Fig. 6:**
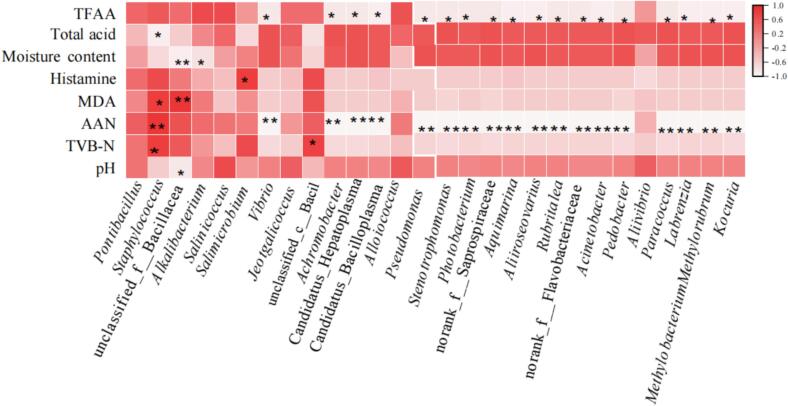
A heatmap depicting the relationship between the physicochemical characteristics and the genera of low-salt shrimp paste. (* represents *p* < 0.05; ** represents *p* < 0.01).

Fig. 6 depicts the relationship between the physical and chemical properties and microorganism genus during the fermentation of low-salt shrimp paste. The findings demonstrated that the bacterial genera *Staphylococcus*, unclassified_f__Bacillaceae, *Vibrio*, and unclassified_c__Bacilli are associated with physicochemical features. *Staphylococcus* plays an important role in physicochemical properties and is significantly positively associated with TVB-N, AAN, and MAD, the water content and total acid are significantly negatively correlated. The *Staphylococcus* has high protease activity, which promotes the breakdown of proteins in shrimp paste, resulting in higher levels of TVB-N and AAN in the shrimp paste. *Salimicrobium* showed a significant positive correlation with histamine (*p* < 0.05). The unclassified_f__Bacillaceae showed a strong positive correlation with MDA (*p* < 0.01) and a substantial negative correlation with pH value(*p* < 0.05) and the water content (*p* < 0.01). Unclassified_f__Bacillaceae may influence the levels of MDA and pH through their metabolic activities, particularly in oxidation reactions and acid-base regulation processes. *Vibrio* is significantly negatively correlated with AAN (*p* < 0.01) and TFAA (*p* < 0.05). Unclassified_c__Bacilli has a significant positive correlation with TVB-N (*p* < 0.05). *Achromobacte,* Candidatus_Hepatoplasma, Candidatus_Bacilloplasma, *Pseudomonas*, *Stenotrophomonas*, *Photobacterium*, norank_f__Saprospiraceae, *Aquimarina*, *Aliiroseovarius*, *Rubritalea*, norank_f__Flavobacteriaceae, *Acinetobacter*, *Pedobacter*, *Paracoccus*, *Labrenzia*, *Methylobacterium-Methylorubrum*, and *Kocuria* were all significantly negatively correlated with AAN (*p* < 0.01). *Vibrio*, *Achromobacter*, Candidatus Hepatoplasma, Candidatus Bacilloplasma, *Pseudomonas*, *Stenotrophomonas*, *Photobacterium*, norank_f__Saprospiraceae, *Aquimarina*, *Aliiroseovarius*, *Rubritalea*, norank_f__Flavobacteriaceae, *Acinetobacter*, *Pedobacter*, *Paracoccus*, *Labrenzia*, *Methylobacterium*, *Methylorubrum*, and *Kocuria* are significantly negatively correlated with TFAA (*p* < 0.05). In general, bacteria greatly influenced the physicochemical properties of low-salt shrimp paste during fermentation.

## 4 Conclusions

This study evaluated the alterations in physicochemical characteristics and microbiological diversity throughout the fermentation process of low-salt shrimp paste by including glucose (P) and glutinous rice flour (F). The physicochemical properties of control (Y), P, and F groups changed gradually with the increase in fermentation time. Throughout the fermentation process, the low-salt shrimp paste with glucose and glutinous rice flour had lower TVB-N values and higher AAN values, which increased the flavor of the shrimp paste compared to the control group, and reduced the possibility of food spoilage. The addition of glucose and glutinous rice flour to low-salt shrimp paste is beneficial in inhibiting histamine production. The microbial community's composition changes at various stages of fermentation, which in turn influences the quality of low-salt shrimp paste. Bacillaceae were partially pathogenic and the addition of glucose and glutinous rice flour reduced the relative abundance of Bacillaceae. Correlation analysis showed that the changes in physicochemical properties were associated with a variety of microorganisms, together affecting the quality of low-salt shrimp paste. This study provides a theoretical reference for improving the quality of shrimp paste, reducing the cost of shrimp paste, and further advancing the fermentation process of novel low-salt shrimp paste.

## CRediT authorship contribution statement

**Meiqi Gu:** Writing – original draft, Investigation, Conceptualization. **Ting Li:** Investigation, Conceptualization. **Pinghui Pan:** Conceptualization. **Aneth Massawe:** Investigation, Conceptualization. **Chuandong Fang:** Investigation, Conceptualization. **Chuanhai Tu:** Writing – review & editing, Supervision. **Zhiyu Liu:** Supervision, Investigation. **Bin Zhang:** Supervision, Investigation.

## Declaration of competing interest

The authors declare that they have no known competing financial interests or personal relationships that could have appeared to influence the work reported in this paper.

## Data Availability

The data that has been used is confidential.
